# Comparison of the Characteristics of Coronary Interventions Performed During Day and Night Shifts in Patients with Acute Myocardial Infarction

**DOI:** 10.3390/ijerph17155378

**Published:** 2020-07-26

**Authors:** Michał Chyrchel, Tomasz Gallina, Oskar Szafrański, Łukasz Rzeszutko, Andrzej Surdacki, Stanisław Bartuś

**Affiliations:** 1 Second Department of Cardiology, Jagiellonian University Medical College, 2 Jakubowskiego Street, 30-688 Cracow, Poland; mchyrchel@gmail.com (M.C.); lrzeszutko@cathlab.krakow.pl (Ł.R.); mbbartus@cyfronet.pl (S.B.); 2 Department of Cardiology and Cardiovascular Interventions, University Hospital, 2 Jakubowskiego Street, 30-688 Cracow, Poland; 3 Students’ Scientific Group at the Second Department of Cardiology, Jagiellonian University Medical College, 2 Jakubowskiego Street, 30-688 Cracow, Poland; tomasz.gallina@interia.pl (T.G.); osk.sza2@gmail.com (O.S.)

**Keywords:** acute myocardial infarction, percutaneous coronary intervention, procedural characteristics, off-hours intervention, night-time shift, operators’ expertise

## Abstract

Therapeutic percutaneous coronary intervention (PCI) is the treatment of choice in acute myocardial infarction (AMI). If optimally performed, PCI reduces myocardial injury and improves the likelihood of a positive clinical outcome. Therefore, the equal quality of PCI throughout both day and night shifts is of paramount importance. Our aim was to compare urgent diagnostic and therapeutic coronary interventions performed during day and night shifts. We retrospectively analyzed the medical records of 144 patients who underwent coronary angiography for AMI over six months in a tertiary referral center working in 24/7 mode. The patients’ characteristics, procedural data and the operator’s experience in interventional cardiology were compared according to the time of intervention during a day shift (8 a.m. until 8 p.m., group A, *n* = 106) and night shift (from 8 p.m. until 8 a.m. next day, group B, *n* = 36). The baseline characteristics of the subjects of groups A and B were similar, except for a higher proportion of AMI without persistent ST-segment elevation (NSTEMI) in patients who underwent coronary angiography during regular working hours compared to off-hours (58% vs. 34%, *p* < 0.05). The average time of diagnostic coronary angiography was longer by about 5 min during the day shift (28.5 ± 12.2 vs. 23.8 ± 8.9 min, *p* < 0.05), while other procedural data, including the arterial access route, the number of catheters needed and the contrast-medium volume, were similar. The use of additional diagnostic tools for coronary lesion assessment (intracoronary ultrasound or fractional flow reserve measurement) was almost twice as frequent during regular working hours (15% vs. 8%). Urgent therapeutic PCI on the culprit artery was performed in 79% and 89% of group A and B patients, respectively. The groups did not differ in procedural characteristics regarding the total interventional session, including both diagnostic angiography and therapeutic PCI, such as total procedure duration, fluoroscopy time, radiation dose, stenting technique and total stent length. Coronary thrombectomy or rotational atherectomy were more frequently used in group A (27% vs. 15%, *p* = 0.16). The percentage of doctors with the least experience in interventional cardiology was, albeit insignificantly, lower during day shifts (31% vs. 42%). In conclusion, the majority of clinical and periprocedural characteristics appeared to be independent of intervention time, except for a longer duration of diagnostic coronary angiography during daytime. This finding could probably result from a higher proportion of NSTEMI patients frequently requiring additional angiographic projections and special techniques to properly identify the infarct-related artery during the day shift. Whether a tendency of less frequent use of additional tools at off-hours may also be due to a lower percentage of NSTEMI interventions at night, or whether this can be linked to lower availability of experienced operators, remains to be validated in a large study. The latter possibility, if confirmed, might encourage public health authorities and healthcare organizers to improve off-hours cathlab staffing with experienced interventionalists. Finally, additional obligatory training in special diagnostic and therapeutic invasive techniques might be advisable for the least experienced operators scheduled to work night shifts.

## 1. Introduction

Therapeutic percutaneous coronary intervention (PCI) is the treatment of choice in patients with acute myocardial infarction (AMI) [1,2]. The benefits of PCI increase in correlation with the brevity of the time from symptom onset to opening of the infarct-related artery [3]. If optimally performed, PCI reduces myocardial injury and improves the likelihood of a positive clinical outcome [1,2]. Therefore, the equal quality of PCI throughout both day and night shifts is of paramount importance.

There are conflicting data on the short-term efficacy and long-term effects of therapeutic PCI performed outside of regular working hours [4,5,6,7,8,9,10,11]. Some reports suggest that off-hours coronary interventions (at night time and on weekends) could be linked to a lower acute procedural success rate and an excessive risk of complications, which may adversely affect further clinical outcomes [5,8,10,11]. However, no effects regarding the time of intervention were found in other studies [6,7,9]. According to a meta-analysis [4], short-term mortality was higher after off-hours PCI for AMI with persistent ST-segment-elevation (STEMI), which was more pronounced in non-North American studies.

Several factors can influence PCI characteristics according to the time of intervention, including distinct patients’ clinical profiles, operators’ experience and technical procedural factors [4,8,10]. Our aim was to compare the patient-related, operator-dependent and technical characteristics of invasive coronary procedures (both diagnostic and therapeutic) performed for AMI during day and night shifts in a cathlab operating in 24/7 mode.

## 2. Materials and Methods

We retrospectively analyzed the in-hospital medical records of 144 AMI patients urgently referred over six months to a cathlab operating in 24/7 mode. Final diagnoses of AMI were based on the established criteria [1,2]. Patients were divided into two groups according to the time of coronary intervention. Group A (*n* = 106) encompassed AMI subjects undergoing coronary intervention during the day shift (from 8 a.m. until 8 p.m., i.e., regular working hours), whereas group B included patients (*n* = 38) operated on during the night shift (from 8 p.m. until 8 a.m. the next day).

Patients’ characteristics and periprocedural data were retrospectively collected by chart review. Periprocedural data included vascular access, time of procedure, contrast-medium volume, radiation dose, number of diagnostic and therapeutic catheters, number and length of stents and the use of special tools and techniques, i.e., intracoronary ultrasound (ICUS), fractional flow reserve (FFR) assessment, thrombectomy and rotational atherectomy. Clinical decisions following diagnostic coronary angiography were also recorded, i.e., urgent PCI, coronary artery bypass grafting (CABG) and optimal medical therapy (OMT). Operators who performed coronary procedures were classified into four groups based on the extent of their experience in coronary interventions: below 5 years, 5–10 years, 10–15 years and over 15 years.

The ethical committee of our university approved the protocol, including the fact that the patients’ informed consent was not sought, owing to the retrospective design of the study (approval No. 1072.6120.85.2020 issued on 23 April 2020).

Data are shown as means ± standard deviation (SD) or numbers (*n*) with proportions (%). Intergroup comparisons were made by a 2-tailed Student’s t-test for continuous characteristics and Fisher’s exact test (2 × 2 frequency tables) or Chi-square test for categorical data. A *p*-value below 0.05 was considered to be significant.

## 3. Results

STEMI and NSTEMI were diagnosed in 63 and 75 patients, respectively, out of 144 study subjects, while the remaining six AMI patients had ventricular paced rhythm at admission. The time of diagnostic angiography was longer (*p* < 0.05) and contrast-medium volume higher (*p* < 0.01) in NSTEMI patients in comparison to STEMI subjects.

The baseline characteristics of group A and B subjects were similar, except for a higher proportion of NSTEMI during the day compared to night shift (58% vs. 34%, *p* < 0.05) (Table 1). The distribution of significant coronary lesions was also comparable across the groups (Table 1). 

The average time of diagnostic coronary angiography was longer by about 5 min during the day shift (*p* < 0.05), while other procedural data, including the arterial access route, the number of catheters needed and the contrast-medium volume were similar. The use of additional diagnostic tools for coronary lesion assessments (ICUS or FFR measurement) was almost twice as frequent during the day shift (15% vs. 8%). Nonetheless, the intershift difference did not reach the assumed statistical significance (Table 1).

Urgent therapeutic PCI on the infarct-related artery was performed in 84 (79%) and 34 (89%) patients from groups A and B, respectively. The groups did not differ in procedural characteristics regarding the total interventional session, including both diagnostic angiography and therapeutic PCI, such as total procedure duration, fluoroscopy time and radiation dose. In addition, the rate of stenting, stenting technique, the number of stents and total stent length were also comparable (Table 2). However, coronary thrombectomy tended to be more frequent in group A (25% vs. 15%), while rotational atherectomy due to severe calcifications was applied only in two group A subjects (Table 2). In sum, these special therapeutic techniques were used almost twice more frequently in group A compared to group B patients (27% vs. 15%, *p* = 0.16). 

There were no significant intergroup differences in operator expertise; however, the percentage of doctors with the least experience in interventional cardiology was lower, albeit insignificantly, during day shifts (31% vs. 42%) (Table 1). Increased operator experience in invasive cardiology (>10 years) tended to be associated with a shorter duration of diagnostic or therapeutic interventions, irrespective of AMI type or procedure time (day or night shift) (*p* = 0.15).

## 4. Discussion

Our study highlights a longer average duration of diagnostic coronary angiography during the day shift compared to the night shift. This finding may be due to a higher proportion of NSTEMI patients in regular working hours, because more time and additional angiographic projections are usually required to identify the culprit vessel in NSTEMI subjects presenting without ST-segment elevations typical for the infarct-related artery. Indeed, the angiography time and contrast-medium volume were increased in our NSTEMI patients compared to STEMI subjects. 

However, the duration of the total procedural session, including both diagnostic angiography and therapeutic PCI, total fluoroscopy time or radiation doses were similar during day and night shifts. Notably, during day shifts, the percentage of interventions carried out by the least experienced operators (less than 5 years of experience in interventional cardiology) was slightly lower than at night (31% vs. 42%). Accordingly, a greater average operator experience could contribute to the observation that, despite a higher percentage of NSTEMI patients, longer diagnostic angiography and more frequent use of additional tools and special techniques during day shifts, longer diagnostic angiographies did not translate into significant intershift differences in any parameter regarding the total interventional session. Likewise, the type of vascular access, treatment strategy, rate of direct stenting, lesion predilatation and stent optimization with a noncompliant balloon catheter were comparable, regardless of intervention time.

On the other hand, it cannot be excluded that about a two-fold lower use of additional diagnostic methods (ICUS or FFR assessment) (Table 1) and therapeutic techniques (coronary thrombectomy or rotational atherectomy) (Table 2) during off-hours might be due not to a lower percentage of NSTEMI interventions at night, but rather, to the reduced availability of experienced operators. The latter observation, if confirmed by a large study, might require coordinated administrative and organizational efforts to improve off-hour cathlab staffing with experienced invasive cardiologists. It might also be proposed that mandatory training in the use of special techniques, like ICUS, FFR, thrombectomy and rotational atherectomy, might be advisable for the least experienced operators before their first night shift in the cathlab.

In general, the number of AMI patients who undergo urgent coronary intervention is higher during regular hours [5,6,8], which appears secondary to a circadian variation of symptom onset [11], but may also be enhanced by better availability of cathlabs and skilled operators during the day shift. Also, interventions are frequently delayed until morning in NSTEMI patients admitted at night with less pronounced symptoms, only slightly elevated troponin levels and a potential alternative diagnosis, which appears to be a likely basis for the higher proportion of NSTEMI subjects being treated during the day shift in our study group.

Nevertheless, patients presenting out of hours could have more severe clinical symptoms and increased prevalence of multivessel coronary artery disease (CAD) [8] compared to those presenting during routine duty hours, which may explain the elevated short-term risk of adverse CV events [4,5,8,10,11], in addition to longer door-to-balloon time in some studies [4,10]. However, in our hands, the angiographic CAD extent and the number and total length of stents were similar for urgent interventions performed during the day and at night. Additionally, hypotension associated with the need for inotropes or vasopressors was observed in a similar proportion of patients, irrespective of intervention time (Table 1).

Our study was limited by the small number of the subjects and the retrospective single-center design. Importantly, clinical and procedural data were limited to those available in the cathlab medical records. In addition, we defined off-hour interventions as those performed during night shifts, regardless of whether these fell on a weekday or at the weekend. In contrast, in the majority of studies, all weekend interventions were also referred to as occurring outside regular hours [4]. However, in a preliminary analysis of data from our regional hospital network, we observed intershift differences in the same direction in most analyzed parameters for PCI performed at weekends and on regular weekdays.

## 5. Conclusions

The majority of clinical and periprocedural characteristics appear to be independent of the intervention time, except for a longer duration of diagnostic coronary angiography during day shifts. This observation probably results from a higher proportion of NSTEMI patients during the day shift frequently requiring additional angiographic projections and additional diagnostic tools to properly identify the culprit artery. Whether the tendency of less frequent use of additional diagnostic and therapeutic techniques at off-hours may also be due to a lower percentage of NSTEMI interventions at night, or may be linked to the reduced availability of experienced operators, remains to be validated in a larger study. The latter possibility, if confirmed, might encourage public health authorities and healthcare organizers to improve off-hour cathlab staffing with experienced interventionalists. Finally, additional obligatory training in special diagnostic and therapeutic invasive techniques might be advisable for the least experienced operators scheduled to work night shifts.

## Figures and Tables

**Table 1:** Basic patient characteristics, procedural data and operator experience.

Characteristic	Day Shift (*n* = 106)	Night Shift (*n* = 38)	*p*-Value
Basic patient characteristics			
Gender, men/women, *n* (%)	74/32 (70/30)	24/14 (63/37)	0.5
Age, years	69 ± 12	68 ± 11	0.7
Diagnosis, STEMI/NSTEMI, *n* (%)	42/62 (40/58)	21/13 (55/34)	0.046
Need of inotropes or vasopressors, *n* (%)	12 (11)	5 (13)	0.8
Arterial access route			
Initial access site, RRA/LRA/FA, *n* (%)	84/7/15 (79/7/14)	32/4/2 (84/11/5)	0.3
Change of access site, *n* (%)	10 (9)	2 (5)	0.7
Diagnostic coronary angiography			
Duration, min	28.5 ± 12.2	23.8 ± 8.9	0.03
Contrast-medium volume, mL	78 ± 27	70 ± 26	0.12
Number of catheters used	1.6 ± 0.6	1.6 ± 0.6	0.9
Additional diagnostic tools (ICUS or FFR), *n* (%)	16 (15)	3 (8)	0.4
Treatment strategy, PCI/CABG/OMT	84/5/17 (79/5/16)	34/1/3 (89/3/8)	0.4
Distribution of significant coronary lesions			0.9
Left main coronary artery, *n* (%)	12 (11)	4 (10)	
Left anterior descending artery, *n* (%)	62 (58)	25 (66)	
Left circumflex coronary artery, *n* (%)	42 (40)	13 (34)	
Right coronary artery, *n* (%)	51 (48)	20 (53)	
Operator expertise (years of experience in interventional cardiology)			0.7
<5 years, *n* (%)	33 (31)	16 (42)	
5‒10 years, *n* (%)	35 (33)	10 (26)	
10‒15 years, *n* (%)	11 (10)	4 (11)	
>5 years, *n* (%)	27 (25)	8 (21)	

Data are shown as mean ± SD or numbers (%). CABG: coronary artery bypass grafting; FA: femoral artery; FFR: fractional flow reserve; ICUS: intracoronary ultrasound; LRA: left radial artery; NS: nonsignificant; NSTEMI: myocardial infarction without persistent ST-segment elevation; OMT: optimal medical therapy; PCI: urgent percutaneous coronary intervention on the culprit artery; RRA: right radial artery; STEMI: myocardial infarction with persistent ST-segment elevation.

**Table 2:** Procedural characteristics of patients undergoing PCI for AMI.

Characteristic	Day Shift (*n* = 84)	Night Shift (*n* = 34)	*p*-Value
Total procedural session (both angiography and PCI)			
Procedure duration, min	69.8 ± 28.6	62.5 ± 26.3	0.2
Time of fluoroscopy, min	14.0 ± 9.4	14.1 ± 10.0	0.9
Radiation dose, Gy	0.7 ± 0.6	0.8 ± 0.6	0.4
Contrast-medium volume, mL	168 ± 77	167 ± 66	0.9
Therapeutic PCI on the culprit artery			
Stent implantation, *n* (%)	70 (84)	31 (91)	0.4
Direct stenting, *n* (%)	7 (8)	5 (15)	0.3
Lesion pre-dilatation, *n* (%)	59 (70)	26 (76)	0.4
Stent post-dilatation with a noncompliant	50 (60)	25 (74)	0.2
balloon, *n* (%)			
Number of stents, *n*	1.4 ± 0.7	1.4 ± 0.8	0.9
Total stent length, mm	32 ± 22	31 ± 18	0.8
Coronary thrombectomy, *n* (%)	21 (25)	5 (15)	0.3
Rotational atherectomy, *n* (%)	2 (2)	0 (0)	1

Data are shown as mean ± SD or numbers (%). AMI: acute myocardial infarction; PCI: percutaneous coronary intervention.
